# Public health policies and health-care workers’ response to the COVID-19 pandemic, Thailand

**DOI:** 10.2471/BLT.20.275818

**Published:** 2021-02-01

**Authors:** Natthaprang Nittayasoot, Rapeepong Suphanchaimat, Chawetsan Namwat, Patcharaporn Dejburum, Viroj Tangcharoensathien

**Affiliations:** a Department of Disease Control, Ministry of Public Health, Bangkok, Thailand.; b International Health Policy Programme, Ministry of Public Health, Tivanond Road, Nonthaburi 11000, Thailand.

## Abstract

Since January 2020, the coronavirus disease 2019 (COVID-19) pandemic has had a far-reaching impact on global morbidity and mortality. The effects of varying degrees of implementation of public health and social measures between countries is evident in terms of widely differing disease burdens and levels of disruption to public health systems. Despite Thailand being the first country outside China to report a positive case of COVID-19, the subsequent number of cases and deaths has been much lower than in many other countries. As of 7 January 2021, the number of confirmed COVID-19-positive cases in Thailand was 9636 (138 per million population) and the number of deaths was 67 (1 per million population). We describe the nature of the health workforce and function that facilitated the capacity to respond to this pandemic. We also describe the public health policies (laboratory testing, test-and-trace system and mandatory 14-day quarantine of cases) and social interventions (daily briefings, restriction of mobility and social gatherings, and wearing of face masks) that allowed the virus to be successfully contained. To enhance the capacity of health-care workers to respond to the pandemic, the government (i) mobilized staff to meet the required surge capacity; (ii) developed and implemented policies to protect occupational safety; and (iii) initiated packages to support morale and well-being. The results of the policies that we describe are evident in the data: of the 66 countries with more than 100 COVID-19-positive cases in health-care workers as at 8 May 2020, Thailand ranked 65th.

## Résumé

Depuis janvier 2020, la pandémie de maladie à coronavirus (COVID-19) a eu un impact considérable sur la morbidité et la mortalité à l'échelle globale. Les degrés de mise en œuvre des mesures sociales et sanitaires, qui varient d'un pays à l'autre, ont des conséquences évidentes, notamment sur les différences de charge que représente la maladie et sur l'ampleur des perturbations touchant les systèmes de santé publique. Même si la Thaïlande est, après la Chine, la première nation à avoir signalé un cas positif de COVID-19, le nombre de cas et de décès qui ont suivi a été nettement moins élevé que dans de nombreux autres pays. Au 7 janvier 2021, la Thaïlande comptait 9636 cas positifs confirmés de COVID-19 (138 par million d'habitants) et 67 décès (1,0 par million d'habitants). Dans le présent document, nous décrivons la nature des professionnels de santé et des fonctions qui ont renforcé les capacités de réaction face à cette pandémie. Nous détaillons également les politiques de santé publique (tests en laboratoire, système de dépistage et de suivi, quarantaine obligatoire de 14 jours pour les cas détectés) et les interventions sociales (séances d'information quotidiennes, restriction des déplacements et des rassemblements, port du masque) qui ont permis de contenir le virus avec succès. Afin d'aider les soignants à lutter contre la pandémie, le gouvernement (i) a mobilisé du personnel pour fournir les capacités d'intervention requises; (ii) a développé et appliqué des mesures de protection pour garantir la sécurité au travail; et enfin, (iii) a proposé des programmes de soutien au moral et au bien-être. Les politiques que nous évoquons se traduisent par des résultats sans équivoque: sur les 66 pays dépassant les 100 cas positifs de COVID-19 chez les professionnels de santé au 8 mai 2020, la Thaïlande se classait à la 65^e^ place.

## Resumen

Desde enero de 2020, la pandemia de la enfermedad por coronavirus (COVID-19) ha tenido un impacto de gran alcance en la morbilidad y la mortalidad mundial. Los efectos de los diferentes grados de aplicación de las medidas sociales y de salud pública entre los países son evidentes en términos de cargas virales muy diferentes y niveles de perturbación de los sistemas de salud pública. A pesar de que Tailandia fue el primer país fuera de China en notificar un caso positivo de COVID-19, el número posterior de casos y muertes ha sido mucho menor que en muchos otros países. Hasta el 7 de enero de 2021, el número de casos positivos confirmados de COVID-19 en Tailandia era de 9.636 (138 por millón de población) y el número de muertes era de 67 (1,0 por millón de población). Describimos la naturaleza del personal sanitario y la función que facilitó la capacidad de respuesta a esta pandemia. También describimos las políticas de salud pública (pruebas de laboratorio, sistema de prueba y rastreo y cuarentena obligatoria de 14 días), así como las intervenciones sociales (sesiones informativas diarias, restricción de la movilidad y de las reuniones sociales, uso de mascarillas, etc.) que permitieron contener el virus con éxito. Para mejorar la capacidad de los trabajadores sanitarios para responder a la pandemia, el gobierno (i) movilizó al personal para satisfacer la capacidad de respuesta requerida; (ii) desarrolló y aplicó políticas para proteger la seguridad laboral; y (iii) puso en marcha paquetes para apoyar la moral y el bienestar de la población. Los resultados de las políticas que describimos son evidentes en los datos: de los 66 países con más de 100 casos positivos de COVID-19 en trabajadores sanitarios a 8 de mayo de 2020, Tailandia ocupaba el puesto 65.

## ملخص

منذ يناير/كانون ثاني 2020، كان لجائحة مرض فيروس كورونا (كوفيد 19) تأثيرًا بعيد المدى على معدلات المرض والوفيات العالمية. إن آثار الدرجات المتفاوتة لتنفيذ تدابير الصحة العامة والتدابير الاجتماعية بين البلدان، ظهرت جلية في صورة أعباء المرض المتفاوتة بقوة، ومستويات الاضطراب في نظم الصحة العامة. على الرغم من أن تايلند هي أول دولة خارج الصين تبلغ عن حالة إيجابية لكوفيد 19، إلا أن العدد اللاحق للحالات والوفيات كان أقل بكثير من العديد من الدول الأخرى. بدءًا من 7 يناير/كانون ثاني 2021، كان عدد الحالات الإيجابية المؤكدة لجائحة كوفيد 19 في تايلند هو 9636 حالة (138 حالة لكل مليون نسمة)، وكان عدد الوفيات هو 67 حالة (1.0 حالة لكل مليون نسمة). نحن نصف طبيعة ووظيفة القوى العاملة الصحية التي سهلت القدرة على الاستجابة لهذه الجائحة. كما قمنا كذلك بوصف السياسات الصحية العامة (الاختبارات المعملية، ونظام الاختبار والتتبع، والحجر الصحي الإلزامي لمدة 14 يومًا للحالات)، والتدخلات الاجتماعية (الموجزات اليومية، وتقييد التنقلات والتجمعات الاجتماعية، وارتداء أقنعة الوجه)، التي سمحت باحتواء الفيروس بنجاح. لتعزيز قدرة العاملين في مجال الرعاية الصحية على الاستجابة للجائحة، قامت الحكومة (1) بحشد فريق العمل لتلبية الاحتياجات المفاجئة المطلوبة؛ و(2) تطوير وتنفيذ سياسات لحماية السلامة المهنية؛ و(3) بدأت حزم لدعم الروح المعنوية والرفاهية. إن نتائج السياسات التي نقوم بوصفها، تظهر بجلاء في البيانات: من بين 66 دولة بها أكثر من 100 حالة إيجابية للإصابة بكوفيد 19 في العاملين في مجال الرعاية الصحية حتى 8 مايو/أيار 2020، احتلت تايلند المرتبة 65.

## 摘要

自 2020 年 1 月以来，冠状病毒病（新型冠状病毒肺炎）大流行在全球范围内造成了极高的发病率和死亡率。本次大流行对各国造成的疾病负担以及对其公共卫生系统的破坏程度大不相同，从这个角度看来，各国不同的公共卫生和社交措施实施程度所产生的影响非常明显。尽管泰国是中国境外第一个报告新型冠状病毒肺炎阳性病例的国家，但该国随后的病例数量和死亡人数却大大低于其他许多国家。截至 2021 年 1 月 7 日，泰国的新型冠状病毒肺炎阳性确诊病例累计为 9636 例（占百万分之 138），死亡人数为 67 例（占百万分之 1.0）。我们描述了卫生部门人力资源和职能的性质（其性质导致泰国应对该大流行的能力得到加强）。我们还描述了促进成功控制该病毒的公共卫生政策（实验室检测、检测与跟踪系统以及强制隔离 14 天）和社交干预措施（每日情况通报、人员流动和社交聚会限制措施以及佩戴口罩防护措施）。为了增强医护人员应对大流行的能力，政府 (i) 动员医护人员参与抗疫以满足激增的需求；(ii) 制定并实施政策以保障职业安全；(iii) 发起一揽子计划以鼓舞士气和促进身心健康。通过数据可以发现，上述政策所取得的成绩显而易见：截至 2020 年 5 月 8 日，在新型冠状病毒肺炎阳性医护工作者数量超过 100 例的 66 个国家中，泰国排名第 65 位。

## Резюме

С января 2020 года пандемия коронавирусного заболевания (COVID-19) оказала масштабное влияние на глобальную заболеваемость и смертность. Эффекты различной степени от реализации мер общественного здравоохранения и социальных мер в разных странах очевидны с точки зрения сильно различающегося бремени болезней и уровней дезорганизации систем общественного здравоохранения. Хотя Таиланд и является первой страной за пределами Китая, сообщившей о положительном случае COVID-19, однако последующее число случаев заболевания и смертей было намного ниже, чем во многих других странах. По состоянию на 7 января 2021 года количество подтвержденных случаев заражения COVID-19 в Таиланде составило 9636 (138 случаев на миллион населения), а количество смертей — 67 (1,0 случая на миллион населения). Авторы описывают характер действий кадровых ресурсов здравоохранения и функции, которые поддерживали способность реагировать на эту пандемию. Также описываются политики общественного здравоохранения (лабораторное тестирование, система тестирования и отслеживания и обязательный 14-дневный карантин случаев) и социальные вмешательства (ежедневные брифинги, ограничение мобильности и общественных собраний, ношение масок для лица), которые позволили успешно сдерживать вирус. Чтобы повысить способность работников здравоохранения реагировать на пандемию, правительство (i) мобилизовало персонал для обеспечения необходимого резерва; (ii) разработало и реализовало политики по охране труда; (iii) инициировало программы для поддержки морального духа и благополучия. Результаты описываемой политики очевидны в рамках данных: из 66 стран, в которых по состоянию на 8 мая 2020 года было зарегистрировано более 100 случаев заражения COVID-19 среди работников здравоохранения, Таиланд занял 65-е место.

## Introduction

Wuhan Municipal Health Commission, China, reported a cluster of cases of pneumonia of unknown etiology, later named coronavirus disease 2019 (COVID-19), on 31 December 2019.^1^ In response to this threat, the Thailand Ministry of Public Health set up an Emergency Operations Centre on 4 January 2020 to provide daily technical support and advice to the government,^2^ and the first COVID-19-positive case outside China was reported in Thailand on 13 January 2020. Epidemiological evidence shows that the index cases were all diagnosed in non-Thai travellers who entered Thailand before international travel restrictions were enforced.^3^ These initial cases resulted in the transmission of severe acute respiratory syndrome coronavirus-2 (SARS-CoV-2) within communities, and the number of new cases peaked in March 2020. Early transmission of the virus was boosted by three clusters of super-spreaders linked to a boxing stadium and to night clubs in Bangkok, and to Muslim pilgrims returning to a few southern provinces from neighbouring countries. 

The Thai government established the Centre for COVID-19 Situation Administration, chaired by the Prime Minister, on 12 March 2020 to harmonize and synergize the government response to the pandemic. By May 2020, the government had achieved containment of the virus through public health and social measures. No local transmission was reported from 25 May until several Thai workers illegally crossed the border into Thailand from Myanmar on 7 November 2020.^4^

Despite being the first country outside China to report a positive case of COVID-19, the subsequent number of cases and deaths has been much lower in Thailand than in many other countries. As of 7 January 2021, the number of confirmed COVID-19-positive cases in Thailand was 9636 (138 cases per million population) and the number of deaths caused by the virus was 67 (1 death per million population; case fatality: 0.7%).^5^ In comparison, the three most-affected countries at that same date were: the United States of America, with 22.1 million cases and 374 133 deaths; India, with 10.4 million cases and 150 606 deaths; and Brazil, with 8.0 million cases and 200 498 deaths. We review the government policies that enabled early containment to be achieved and that enhanced the capacity of health-care workers to provide an effective response to the pandemic.

## Public health workforce 

Thailand’s successful implementation of universal health coverage (UHC), which began in 2002, demonstrates the value of long-term investment in health systems and primary health care.^6^ To accommodate the rapid increase in service utilization required for the implementation of UHC, the Thai government more than doubled the number of qualified nurses and midwives from 84 683 (13.2 per 10 000 population) to 191 575 (27.6 per 10 000 population; 94.8% women) between 2002 and 2018.^7^^,^^8^ During the same period, the government implemented policies to almost treble the number of qualified medical doctors from 18 947 (3.0 per 10 000 population) to 55 890 (8.1 per 10 000 population; 44.7% women).^7^^,^^9^ However, despite significant progress, the combined population density of doctors, nurses and midwives in 2018 (35.7 per 10 000 population) was still lower than the sustainable development goal (SDG) target 3.c of 44.5 per 10 000 population; efforts to achieve the SDG target health workforce density are ongoing.^10^

To address the previously uneven geographical distribution of health-care workers, the government applied multiple interventions such as: increased training capacity; mandatory (since 1972) rural service by graduate doctors, nurses, pharmacists and dentists; the recruitment of health students from rural backgrounds; a training curriculum that included rural health problems; and financial and non-financial incentives such as social recognition.^11^ These interventions, combined with the application of task shifting (the process of delegation of certain tasks, where appropriate, to less-specialized health workers, e.g. nurse practitioners, dental nurses and pharmacy assistants),^12^ mean that the geographical distribution of the health workforce has gradually become more equitable.^13^

Thailand is self-reliant in health-care workforce training, both under- and post-graduate, and all health-care workers are qualified to a high standard.^14^Quality is ensured through the national and continual assessment of all cadres of health-care workers; professional medical councils award the relevant qualifications (licenses), and licenses are maintained by the mandatory completion of a sufficient volume of continued professional education within every five-year period.^15^

## Public health function

Although there is no global consensus on the exact nature of public health, a few key functions identified by existing public health frameworks include: surveillance, governance and financing, health promotion, health protection and legislation, research and human resources.^16^ Public health in Thailand is focused on surveillance, prevention and control, and is fully supported by laboratory and human resources. This definition of public health has been fully integrated at the primary health-care level; district hospitals and health centres provide first-contact services to the entire population.^17^

Disease surveillance has been a function of public health since the inception of the Thai Epidemiology Division in 1970. The first Surveillance and Rapid Response Team was established in 2004, expanding to become a national network of epidemiologists, public health officers and nurses. The teams are responsible for surveillance, outbreak investigations and containment of infectious diseases such as dengue, acute flaccid paralysis, measles, the Zika virus and food poisoning; a total of 87 notifiable diseases were reported in the Weekly Epidemiological Surveillance Report in 2020.^18^ This resilience^19^^,^^20^facilitates the capacity to respond to a large public health emergency or pandemic, for example, the avian influenza pandemic in 2004^21^^,^^22^and the Middle East respiratory syndrome coronavirus in 2015.^23^ The Surveillance and Rapid Response Teams have been the main contributors to public health function since the beginning of the COVID-19 pandemic; in 2020, Thailand had around 1000 such teams distributed across the public health ministry, the provincial health offices and all district hospitals.^24^

Further, in recognizing the interaction between humans, animals and wildlife,^25^ as well as the need for collaboration between medical doctors, veterinarians working with domestic animals and wildlife, and pharmacists, Thailand launched its 3-year field epidemiology training programme in 1980. The World Health Organization recommends an optimal workforce density of one trained field epidemiologist or equivalent per 200 000 population.^26^ Although there are only 183 trained field epidemiologists, equivalent to 0.55 per 200 000 population, in Thailand, the shortfall is being met by on-the-job training delivered to public health officers and nurses. 

## COVID-19 containment

The successful containment of the virus is essential to minimize the additional burden faced by hospitals, prevent health facilities from becoming overwhelmed and sustain the provision of other essential health services. From January 2020 the Thai government implemented several public health interventions to contain the virus, including detection of index cases through laboratory testing and a test-and-trace system to identify all high-risk (i.e. those who have experienced direct contact with respiratory secretions from a COVID-19-positive case) and low-risk contacts. Because voluntary self-isolation at home is not considered to be effective in interrupting transmission, 14-day quarantine at local (i.e. public dormitories or re-purposed sports amenities for Thai citizens in the provinces) or state (i.e. mostly hotels affiliated to hospitals for tests and referrals, for both Thai and non-Thai international travellers) facilities is mandatory for all cases as well as high-risk contacts. 

The government mobilized health-care workers, mostly nurses and public health officers, to support the collection of nasal swabs from all Thai and non-Thai travellers at points of entry (air, land and sea ports) for laboratory analysis, as well as history-taking for the test-and-trace system. Workers were also mobilized to manage, supervise and provide services to contacts of cases at the 14-day quarantine sites. These services included daily clinical monitoring, specimen collection for laboratory testing (at days 3–5 and 11–13) and referral of all positive cases to hospital according to the national protocol.

Contact tracing is facilitated by mandatory registration on the Thai Chana mobile application (app) for everyone visiting a public venue, such as a restaurant or supermarket, or using public transport. The app records name and phone number for tracing if an index case is identified. The app traced 394 contacts in an incident on 10 July 2020, when a non-Thai index case violated regulations by visiting a shopping mall in Rayong province. All contacts were tested and quarantined for 14 days.

Clinicians, in particular critical care specialists, play an important role in the recovery of severely ill patients. Because of the limited feasibility of quickly mobilizing intensive care unit staff from relatively unaffected provinces to where they are urgently needed, all hospitals must always be prepared for an unpredictable epidemic. All public and private facilities with critical care capacity, such as intensive care beds and airborne infection isolation rooms (defined as having negative pressure, 6–12 air exchanges per hour, and a direct exhaust or high-efficiency particulate-air filter to the outdoors),^27^ are required to provide services, and health-care staff protect themselves by adhering to strict protocols. 

The public health ministry instigated the relocation of acute respiratory infection patients to newly constructed shelter units outside the main hospital buildings to reduce their risk of contracting the virus. The ministry also developed standard operating procedures for all health facilities, such as management protocols for the acute respiratory infection clinic and wards containing less severely ill patients, as well as guidelines for the disinfection of all health-care settings.^28^

## Social interventions 

Transparency builds trust and ensures compliance with social interventions. The Centre for COVID-19 Situation Administration has therefore communicated risk and engaged communities in its daily broadcast on all media channels since the beginning of the pandemic. The briefings consist of: an epidemiological update of the regional, national and global situation; the numbers of deaths and positive laboratory tests per million population; and the preventive measures that citizens are required to adopt. 

Government policy to stay at home and work from home in April 2020 restricted the mobility of the population and contributed to the interruption of the virus transmission. In parallel, the government enforced the closure of public venues and banned social gatherings; security officers were responsible for monitoring and supporting adherence to these regulations. 

A systematic review and meta-analysis has shown that physical distancing and the wearing of face masks are also effective in interrupting the transmission of SARS-CoV-2.^29^ Face masks protect others from speech-generated infected droplets from asymptomatic individuals.^30^ The high proportions of asymptomatic positive cases reported – for example, 50–75% in Italy^31^ and 78% in China^32^ – support the wearing of masks to prevent transmission.^33^ The Thai government’s evidence-based strong recommendations have therefore included the wearing of a face mask, practising hand hygiene using alcohol gel, practising food hygiene by not sharing eating utensils or drinking vessels, and physical distancing. Although these measures are not mandatory, adherence by the general population is high; a local survey conducted during April 2020 reported that > 90% of the population were following recommendations regarding the wearing of face masks.^34^

Private sector construction of a new factory in just one month for the local manufacture and free distribution of N95 face masks (i.e. masks that filter at least 95% of airborne particles) to health-care facilities and the general public helped to meet the early demand for face marks. Local government departments mobilized communities and volunteers to produce multilayer cloth masks. Although of lower efficacy,^35^ using cloth masks creates awareness and encourages respiratory hygiene. By the end of July 2020 there were 28 surgical mask factories operating in Thailand, producing 4.2 million masks daily.^36^

The implementation of UHC ensures all Thai and non-Thai members of the public have access to prevention and curative services.^37^ Treatment for affected Thai citizens is financed by their respective insurance schemes; an additional government budget finances the treatment costs of all non-Thai patients, ensuring that there is no financial burden to anyone. The government allocated additional funding to enhance the capacity of certified laboratories in all provinces, provide laboratory testing, and cover the costs of food and lodging at 14-day quarantine sites for all Thai and non-Thai positive cases. The cost of active case detection using laboratory tests among high-risk and vulnerable communities, such as migrant workers, is also fully covered by the government.

## Enhancement of response

To enhance the capacity of health-care workers to respond to COVID-19 and to protect all such workers from infection, three synergistic approaches were implemented from January 2020.

### Surge capacity

A shortage of specialists, in particular intensive care nurses and critical care experts, became evident at the peak of epidemic. Some hospitals deployed experienced nurses from non-intensive care units within their own hospital or province to support the intensive care unit through on-the-job training. In provinces with a high case load and a critical shortage of health-care workers, medical teams were mobilized from other provinces.^38^ The public health ministry closely monitored the pandemic at a provincial level and managed the reallocation of resources.

At the peak of the pandemic in March 2020, all hospitals offered only essential emergency services. Clinical services for well-controlled noncommunicable diseases were transferred to primary care centres at a subdistrict level, protecting patients from the risk of potential infection during a hospital visit. These clinical services were supported by remote consultations and the dispensing of medicines by the postal service^39^ or private pharmacies. Such actions minimized the routine workload of health-care workers, allowing them to direct their resources towards treatment of patients diagnosed with COVID-19.

To support its huge workload, the Department of Disease Control mobilized experienced medical personnel and epidemiologists from provinces with any surplus capacity. The government deployed doctors, nurses and other health personnel to support quarantine sites with suspected positive cases. Local administrations mobilized one million existing village health volunteers to boost the capacity of the Surveillance and Rapid Response Teams for contact tracing. The new volunteers, recruited from local communities by the village head and the existing volunteers, received 43 hours of public health ministry-funded training in the district health office delivered by local public health personnel.^40^ In sharing the dialect, religion and sociocultural practices of local communities, village health volunteers were invaluable in challenging circumstances such as in the southern provinces, where many Muslim pilgrims were returning from other countries. 

### Occupational safety

The Department of Disease Control developed guidelines^41^ recommending that each hospital designate a team of health-care workers specifically for the COVID-19 ward, disallowing rotation to other wards. In some hospitals with severely ill COVID-19 patients, medical teams are divided into two groups – work and off-work, swapping over every 14 days – in case members of one team become infected and require 14-day quarantine. 

The public health ministry is also responsible for ensuring the occupational safety of health-care workers by providing adequate supplies of all types of personal protective equipment. However, public demand for personal protective equipment rose sharply in March 2020, leading to critical shortages in health facilities. After the publication of research demonstrating that sterilization of masks by ultraviolet radiation killed SARS-CoV-2,^42^ some health facilities recycled surgical masks. Plastic raincoats were used as personal protective equipment instead of surgical gowns for the screening of low-risk patients. 

Isolation rooms for airborne infection were engineered by the Siam Cement Group and donated to hospitals for nasal swab and specimen collection^43^ to ensure the occupational safety of the medical team. Hospital staff deployed robots to deliver food and medicines to COVID-19-positive inpatients, and the use of remote communication and monitoring systems protected medical teams from exposure to the virus.

To ensure occupational safety for members of the Surveillance and Rapid Response Teams, all members with high-risk contacts are tested for SARS-CoV-2 and quarantined for 14 days regardless of the test result. Members with low-risk contacts are recommended to self-quarantine and work from home. If a health-care worker becomes infected, an outbreak investigation is conducted immediately to identify the possible source of infection and all contacts are traced for further action. Infection control specialists also developed a safety protocol for the team.

### Morale and well-being

Both the government and the private sector initiated packages to support the morale and well-being of health-care workers. For example, the government approved 40 000 civil servant positions, upgrading all the contract employees, in particular nurses, to civil servant status. In Article 6(4) of the finance ministry 2018 regulation on compensation of health-care workers for adverse events, four types of events (death or permanent disability; loss of organ or disability; infection or serious occupation injury; or infection or injury requiring treatment for less than 20 days) are included. The cabinet approved the doubling of financial compensation to COVID-19-positive health-care workers who required treatment for less than 20 days from 50 000 Thai baht (1670 United States dollars at the time of writing) to 100 000 Thai baht.^44^ An additional allowance per shift was approved for those working in hospitals or quarantine sites. Many insurance companies offered financial protection to all health-care workers against adverse events resulting from the treatment of COVID-19-positive patients in the form of premium-free indemnity coverage.^45^

The prohibition of physical visits or care by family members for dying COVID-19-positive patients, replaced by a virtual presence through telecommunication, causes medical teams significant psychological trauma.^46^^,^^47^ Further, the strict infection control protocol means that family members are not allowed to closely approach or touch the dead body of their relative, a rule that is distressing for both visitors and health-care workers. However, the mental health department provides continual support to health-care workers in the form of a telephone helpline, where health-care workers can speak to qualified psychiatrists or psychologists. 

Finally, health-care workers received national social recognition for their dedication to the pandemic response via the White Gown Hero/Heroine programme that was launched on live television on 29 March 2020 with 5 minutes of applause from citizens.^48^ The public have also been moved to donate food boxes and ready meals to health-care workers on duty at quarantine centres.

## Effect of response

**Fig. 1:**
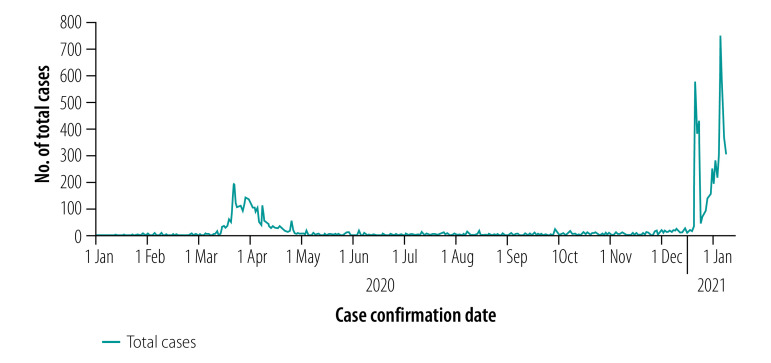
Number of COVID-19 cases among general population, Thailand, 1 January 2020–7 January 2021

**Fig. 2:**
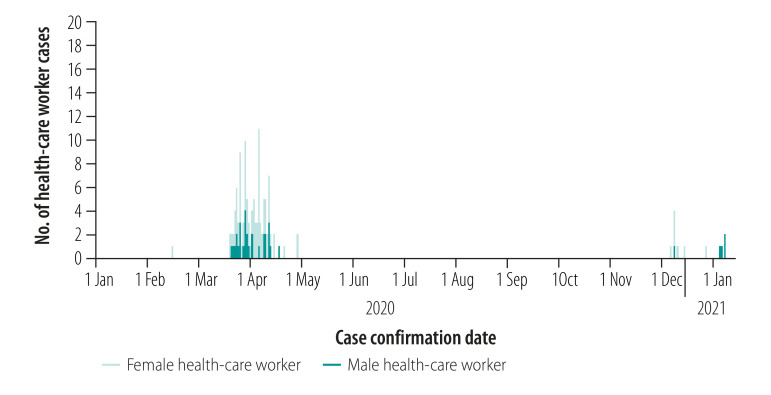
Number of COVID-19 cases among health-care workers, Thailand, 1 January 2020–7 January 2021

Of the 9636 COVID-19-positive cases as at 7 January 2021 (Fig. 1), 122 (1.3%) were health-care workers: 88 (72.1%) women and 34 (27.9%) men (Fig. 2). No health-care workers have died in Thailand as a result of the pandemic. Data collected between 22 July and 15 August 2020 in 37 countries show that the highest numbers of COVID-19-positive cases in health-care workers were reported in the USA (114 529 workers), Mexico (78 200 workers) and Italy (28 896 workers).^49^ The highest numbers of deaths among health-care workers were reported in Mexico (1162 deaths), the USA (574 deaths) and Italy (214 deaths).^49^ Thailand ranked 65th out of 66 countries with more than 100 COVID-19-positive cases in health-care workers as at 8 May 2020.^50^

COVID-19: coronavirus disease 2019.

COVID-19: coronavirus disease 2019

The policies that we have described here indicate that timely interventions minimize mortality. Combined, the function and quality of the Thai public health system, the whole-of-government approach and effective risk communication to the public at the very early stage of the pandemic effectively contained transmission of the virus and prevented the health system from becoming overwhelmed.
